# Nutritional status and impact on outcomes of patients with locally advanced head and neck squamous cell carcinoma treated with concurrent chemoradiotherapy: a pre-planned secondary analysis of a phase 3 randomized controlled trial

**DOI:** 10.1186/s43046-025-00305-y

**Published:** 2025-07-28

**Authors:** Vanita Noronha, Avani Chawda, Vijay Patil, Nandini Menon, Minit Shah, Supriya Goud, Sucheta More, Srushti Shah, Vijayalakshmi Mathrudev, Kumar Prabhash

**Affiliations:** 1 Department of Medical Onclogy, Tata Memorial Hospital, Tata Memorial Center, Homi Bhabha National Institute, Mumbai, Maharashtra India; 2 Department of Medical Oncology, P D Hinduja Hospital & Medical Research Centre, Khar & Mahim, Mumbai, India

**Keywords:** Head-and-neck cancer, Nutrition, Malnutrition, PG-SGA

## Abstract

**Background:**

Adequate nutrition can mitigate side-effects and improve recovery for patients with locally advanced head-and-neck squamous cell cancer (LAHNSCC), while malnourishment can increase morbidity and mortality. We aimed to evaluate the baseline nutritional status of patients with LAHNSCC planned for curative chemoradiotherapy (CRT), the evolution of nutritional status during the course of CRT and to assess whether nutrition impacted their clinical outcomes.

**Methods:**

This was a pre-planned secondary analysis of a Phase III randomized controlled trial conducted between 2013 and 2017 in 300 patients with LAHNSCC who were randomly assigned to receive either cisplatin 30 mg/m^2^ once-a-week or 100 mg/m^2^ once-in-3-weeks concurrently with radiation. This analysis included 112 patients for whom nutritional parameters were recorded. Patient Generated Subjective Global Assessment (PG-SGA) forms were used to evaluate malnutrition severity at different treatment stages. Scores on the PG-SGA ranged from 0 to 35, with higher scores denoting greater malnutrition. Scores were grouped, with 0–3 indicating normal to mild malnutrition, and ≥ 4 denoting moderate to severe malnutrition. Baseline scores were compared with subsequent scores and survival outcomes were analyzed.

**Results:**

At baseline assessment, 42.8% of patients had normal to mild malnutrition, while 57.1% had moderate to severe malnutrition. There were higher baseline malnutrition rates in women, users of smokeless tobacco, and patients with buccal mucosa tumors. By day 21 of treatment, 44 (56.4%) patients had moderate to severe malnutrition, while 34 (43.6%) had normal nutrition or mild malnutrition. Among those with moderate to severe malnutrition at baseline, 13 (29.5%) patients had an improvement in their nutritional status, while 14 (41.2%) patients with normal to mild nutrition at baseline had deterioration in their nutritional status during the course of CRT. Baseline nutritional status did not significantly impact progression-free, locoregional relapse-free or overall survivals.

**Conclusions:**

Pre-treatment nutrition is crucial for managing weight and reducing treatment complications in patients with LAHNSCC. Over 40% of patients with normal baseline nutrition have deterioration of their nutritional status during CRT. We were unable to find any correlation between nutrition and clinical outcomes in patients with LAHNSCC receiving curative CRT. Larger studies are needed to explore the impact of nutrition on treatment outcomes, emphasizing regular dietary assessments and interventions to improve patient compliance.

**Trial registration:**

Clinical Trial Registry of India, under the registration number CTRI/2012/10/003062.

**Supplementary Information:**

The online version contains supplementary material available at 10.1186/s43046-025-00305-y.

## Background

Symptoms and complications stemming from advanced cancer, anticancer treatments, or medical comorbidities can significantly disrupt patients’ appetite and hinder their capacity to consume or metabolize food. These impediments to oral nutritional intake encompass a diverse range of factors termed nutrition impact symptoms. Nutrition impact symptoms such as anorexia, nausea, vomiting, mucositis, and dysphagia can lead to a state of malnutrition [1]. Individuals diagnosed with head-and-neck cancer often face significant challenges associated with chewing and swallowing, which can lead to a state of malnutrition even before the initiation of treatment [2]. This may further cause a reduction in the skeletal muscle mass, which can exacerbate their susceptibility to treatment-related adversities such as reduced treatment tolerance, delayed recovery, impaired immune function, and increased risk of cachexia.

Approximately one-third of patients undergoing radiation therapy experience significant weight loss. The acute toxicities associated with radiation therapy are further intensified when concurrent chemotherapy is administered, potentially leading to exacerbation of symptoms [3]. In severe cases, these treatment-related side-effects may become so debilitating that a decrease in the treatment intensity or cessation becomes necessary [4]. A reduction in treatment intensity adversely affects both the objective response rate and long-term survival. This phenomenon is evident following both radiotherapy and chemotherapy de-escalation [5]. Optimal nutritional status serves as a buffer against adverse effects, diminishing the risk of complications, and improving treatment outcomes, whereas malnourished patients face heightened morbidity and mortality risks.

The Patient-Generated Subjective Global Assessment (PG-SGA), a modified variant of the SGA, serves as a valuable tool for evaluating the nutritional status of patients with cancer [6]. It offers continuous monitoring and facilitates multidisciplinary intervention triage at key treatment junctures. Utilizing numerical scoring, PG-SGA aids in the strategic planning of timely interventions to enhance clinical outcomes [7]. Despite the well-recognized utility of this approach, the nutritional status of Indian patients undergoing chemo-radiotherapy (CRT) for locally advanced head-and-neck squamous cell carcinoma (LAHNSCC) remains underexplored. Hence, we conducted a preplanned analysis focusing on PG-SGA scores in this specific patient cohort.

## Aims and objectives

The primary aim of this study was to evaluate the nutritional status of patients undergoing CRT. The primary objective was to assess if the participants maintained their baseline nutritional parameters during the treatment and post-treatment. The secondary objectives included investigating the correlation between nutritional status and key clinical outcomes such as overall survival, progression-free survival, and loco-regional relapse-free survival in patients with LAHNSCC treated with curative intent CRT.

## Materials and methods

A randomized controlled non-inferiority trial was conducted between 2013 and 2017 at the Department of Medical Oncology of Tata Memorial Hospital, a tertiary care center in Mumbai, India. The study enrolled 300 individuals diagnosed with Stage III or IV non-metastatic squamous cell or undifferentiated carcinoma arising in the oral cavity, oropharynx, hypopharynx, larynx, or cervical adenopathy with unknown primary. Patients were planned for curative intent CRT, either as definitive CRT, or following radical surgery, in patients with high-risk features on pathology (close or positive margins, extracapsular extension, multiple positive lymph nodes, or T4 primary). Among these participants, 150 were allocated to arm A and received cisplatin CRT at 30 mg/m^2^ intravenously once-a-week (started along with radiation and continued until the last day of radiation), and 150 were assigned to arm B, receiving cisplatin CRT 100 mg/m^2^ intravenously once every 3 weeks (on days 1, 22, and 43) [8]. The dose of radiation in the definitive CRT setting was 70 Gy in 35 fractions over 7 weeks; that in the adjuvant setting was 60 Gy. For all patients, radiation was given on 5 days a week, and the dose to the electively treated neck was limited to 46 to 50 Gy, at 2 Gy per fraction, with off-cord portals after 46 Gy. The trial was approved by the institutional ethics committee and was monitored by the institutional data safety and monitoring subcommittee. All patients provided written informed consent. Adherence to ethical guidelines was ensured as per the principles outlined in the 18th Helsinki World Medical Assembly (1964) and its subsequent amendments. The trial was registered with the Clinical Trial Registry of India, under the registration number CTRI/2012/10/003062.

Patients enrolled in the study completed Patient-Generated Subjective Global Assessment (PG-SGA) forms on the 1st, 21st, and 43rd day after initiation of treatment and post-treatment. The first section of the PG-SGA; focusing on weight history, food intake, symptoms, and function, was completed by the patient, with the help of a clinician, if needed. Subsequently, the clinician assessed the disease-stage and factors contributing to metabolic stress, including fever, corticosteroid use, sepsis, and neutropenia. Findings from the physical examination such as loss of subcutaneous fat, muscle wasting, and edema or ascites were noted as well [9].

In this study, PG-SGA-scored nutritional triage recommendations (SNTR) were utilized as an independent assessment scale. Each component of the form was assigned a score ranging from 0 to 35, with higher scores indicating an elevated risk of malnutrition. Triage recommendations were delineated as follows: 0–1: no intervention required with reassessment advised on a routine and regular basis during treatment; 2–3: requirement of a dietitian, nurse, or another clinician to educate the patient and the family about possible pharmacologic interventions; 4–8: requirement of intervention by a dietitian, in conjunction with a nurse or physician as indicated by the symptoms; ≥ 9: indicated a critical need for improved symptom management and/or nutrient intervention options.

These scores were compared between arms A and B and stratified into the above-mentioned categories. Furthermore, scores were aggregated into two groups: 0–3 indicating normal to mild malnutrition, and ≥ 4 denoting moderate to severe malnutrition.

### Statistical analysis

The total sample size of the original study was 300 patients, based on the primary endpoint of locoregional control. For this current analysis, we did not calculate a separate sample size. We included all patients who had completed the PG-SGA forms at baseline and follow-up. The demographic analysis was done by descriptive statistics, counts, percentages, and total scores at different time points between the treatment groups were compared using the chi-square test. The scores were categorized as 0–3 and and ≥ 4, as well as 0–1, 2–3, 4–9, and > 9.

The comparison of categories of scores at each time point with baseline scores was compared using McNemar’s test. Overall survival was defined as the duration between the time of randomization to the date of death or last follow-up. The overall survival (in months) and progression-free survival were calculated using the Kaplan-Meier method [10]. The median follow-up was estimated using the reverse Kaplan-Meier method. The overall survival was compared between the groups by log-rank test. A *P* value of less than 0.05 was considered statistically significant.

## Results

Between 2013 and 2017, 300 patients were enrolled on the study: 150 to the weekly cisplatin arm, and 150 to the high dose 3-weekly cisplatin arm. The results of the study have been earlier published in detail [8]. The majority of patients (279, 93%) received CRT in the adjuvant setting. In arm A, 148 (98.7%) patients started once-a-week cisplatin CRT, and 133 (88.7%) patients completed treatment, while in arm B, 148 (98.7%) started once-in-3-weeks cisplatin CRT and 141 (94%) patients completed their treatment. The median cumulative dose of cisplatin in arm A (weekly cisplatin arm) was 210 mg/m^2^ (IQR, 180–210 mg/m^2^), while that in arm B (3-weekly cisplatin arm) was 300 mg/m^2^ (IQR, 200–300 mg/m^2^).

There were 183 patients who filled out the PG-SGA forms at baseline, but only 112 patients completed the forms at all planned follow-up visits, i.e., on days 22 and 43. The median follow-up duration was 18.9 months (95% confidence interval [CI], 17.1–20.9).

At baseline assessment, 42.8% of patients had normal to mild malnutrition, while 57.1% had moderate to severe malnutrition. Among the cohort, 11 (91.7%) female participants and 53 (53%) male participants were noted to have PG-SGA scores ≥ 4, indicating a higher risk of malnutrition. Similarly, 34 (30.4%) individuals who consumed smokeless tobacco and 66 (58.9%) patients with buccal mucosa primary tumors were disproportionately more likely to present with malnutrition before treatment initiation (Table 1).

**Table 1:** Demographics and pretreatment characteristics of study population categorized as per PG-SGA assessment scores at baseline

**Characteristic**	**0–3 (*n*, %)**	**≥ 4 (*n*, %)**	**Total (%)**	***P* value**
Age group	*n* = 48	*n* = 64	*n* = 112	0.814
18–35	10 (2.1)	13 (20.3)	23 (20.5)	
36–50	26 (54.2)	32 (50)	58 (51.8)	
51–65	12 (25)	18 (28.1)	30 (26.8)	
66–70	0 (0)	1 (1.6)	1 (0.9)	
Gender				0.011
Male	47 (97.9)	53 (82.8)	100 (89.3)	
Female	1 (2.1)	11 (17.2)	12 (10.7)	
Tobacco use				0.002
Non-smoker	22 (45.8)	26 (40.6)	48 (42.9)	
Smokeless tobacco	7 (14.6)	27 (42.2)	34 (30.4)	
Smoking	19 (39.6)	11 (17.2)	30 (26.8)	
Comorbidities				0.663
Hypertension	2 (4.2)	6 (9.4)	8 (7.1)	
Diabetes mellitus	1 (2.1)	2 (3.1)	3 (2.7)	
Hepatitis	0 (0)	1 (1.6)	1 (0.9)	
Cardiac	0 (0)	1 (1.6)	1 (0.9)	
COPD	0 (0)	1 (1.6)	1 (0.9)	
Multiple	2 (4.2)	2 (3.1)	4 (3.6)	
History of head-and-neck cancer	0 (0)	1 (1.6)	1 (0.9)	
Other	1 (2.1)	0 (0)	1 (0.9)	
None	42 (87.5)	50 (78.1)	92 (82.1)	
Site of primary tumor				0.035
Buccal mucosa	23 (47.9)	43 (67.2)	66 (58.9)	
Tongue	17 (35.4)	13 (20.3)	30 (26.8)	
Oropharynx	0 (0)	4 (6.3)	4 (3.6)	
Hypopharynx	0 (0)	1 (1.6)	1 (0.9)	
Larynx	4 (8.3)	2 (3.1)	6 (5.4)	
Cervical lymphadenopathy with unknown primary	4 (8.3)	1 (1.6)	5 (4.5)	
Performance status				0.002
0	2 (4.2)	17 (26.6)	19 (17.0)	
1	46 (95.8)	47 (73.4)	93 (83.0)	
Stage				0.775
III	5 (10.4)	8 (12.5)	13 (11.6)	
IV	35 (72.9)	47 (73.4)	82 (73.2)	

Upon comparing baseline nutrition scores with those at subsequent time points (Table 2), it was observed that a small percentage of patients experienced improvement from their initial scores during each visit and at follow-up. Among patients with baseline scores ≥ 4 (moderate to severe malnutrition), only 13 (29.5%) improved, while 14 (41.2%) patients with baseline scores of 0–3 (normal to mild malnutrition) had deterioration in their nutrition scores. This observed change was not statistically significant.

**Table 2:** Comparison between the total PG-SGA nutritional assessment scores at different time points

**Day**	**Score**	**0**–**3 (%)**	**≥** **4 (%)**	**Total (%)**	***P* value**
Day 21	0**–**3	20 (58.8)	13 (29.5)	33 (42.3)	
	≥ 4	14 (41.2)	31 (70.5)	45 (57.7)	
Total	Count at baseline	34 (43.6)	44 (56.4)	78	1
Day 43	0**–**3	17 (68)	10 (25.6)	27 (42.2)	
	≥ 4	8 (32)	29 (74.4)	37 (57.8)	
Total	Count at baseline	25 (39.1)	39 (60.9)	64	0.815
Follow-up 3	0**–**3	16 (66.7)	11 (30.6)	27 (45)	
	≥ 4	8 (33.3)	25 (69.4)	33 (55)	
Total	Count at baseline	24 (40)	36 (60 %)	60	0.648

**Fig. 1:**
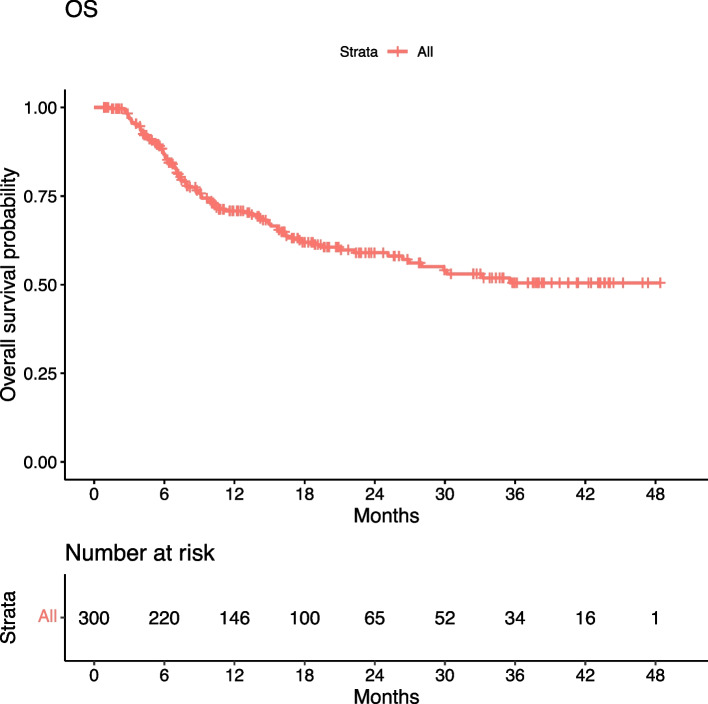
Overall survival

**Fig. 2:**
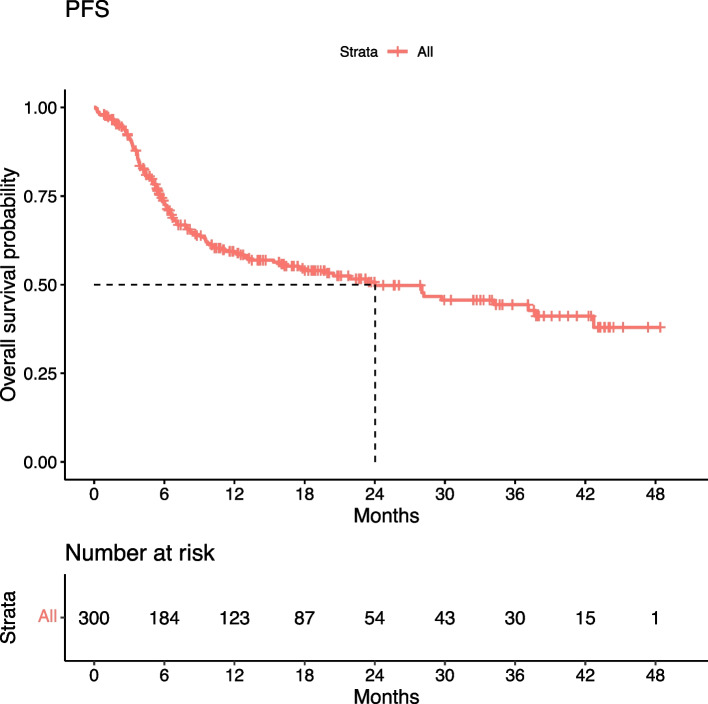
Progression-free survival

**Fig. 3:**
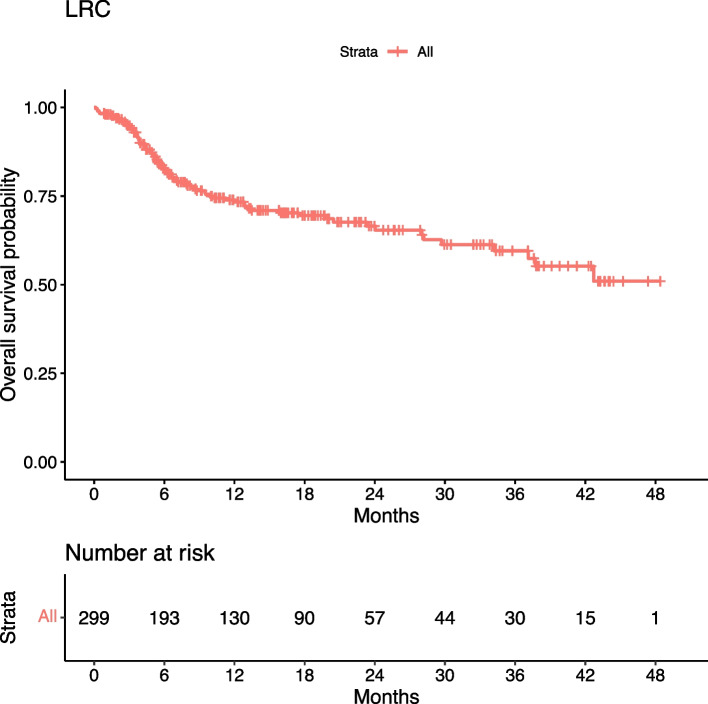
Loco regional relapse-free survival

There was a slightly higher occurrence of events within the group of patients classified as moderately to severely malnourished at baseline. While suggestive of a potential influence of poor baseline nutritional status on outcomes, this observation did not attain statistical significance (Tables 3, 4, and 5). Thus, nutritional status did not appear to significantly impact overall, progression-free, or locoregional relapse-free survivals (Figs. 1, 2, and 3).

**Table 3:** One-year survival rate of participants based on PG-SGA score categories

**Scores**	**Number of participants**	**Number of deaths**	**One-year survival (%)**	***P* value**
Baseline				0.81
0–3	48	13 (27.1%)	74.5	
≥ 4	64	10 (15.6%)	83.3	
Day 21				0.63
0–3	63	17 (27%)	70.6	
≥ 4	68	19 (27.9%)	82.4	
Day 43				0.51
0–3	55	14 (25.5%)	74.8	
≥ 4	59	17 (28.8%)	80.5	
Follow-up 3				0.95
0–3	45	4 (8.9%)	97.6	
≥ 4	49	7 (14.2%)	95.3

**Table 4:** One-year progression-free survival rate based on PG-SGA score categories

**Scores**	**Number of participants**	**Number of events**	**One-year survival (%)**	***P* value**
Baseline				0.23
0–3	48	18 (37.5%)	65.2	
≥ 4	64	23 (35.9%)	69	
Day 21				0.3
0–3	63	19 (30.2%)	68.9	
≥ 4	68	30 (44.1%)	63.8	
Day 43				0.35
0–3	55	20 (36.4%)	60.3	
≥ 4	59	23 (39%)	75.2	
Follow-up 3				0.26
0–3	45	9 (20%)	86.4	
≥ 4	49	11 (22.5%)	90.6

**Table 5:** One-year locoregional relapse-free survival rate based on PG-SGA score categories

**Scores**	**Number of participants**	**Number of events**	**One-year survival (%)**	***P* value**
Baseline				0.86
0–3	48	10 (20.8%)	78.9	
≥ 4	64	17 (26.6%)	75.6	
Day 21				1
0–3	63	12 (19.1%)	77.4	
≥ 4	68	26 (38.2%)	72	
Day 43				0.84
0–3	55	12 (21.8%)	76.1	
≥ 4	59	16 (27.1%)	79.9	
Follow-up 3				0.16
0–3	45	7 (15.6%)	89.6	
≥ 4	49	7 (14.3%)	95.1

## Discussion

In our study, we found that 57% of patients with LAHNSCC were moderately or severely malnourished at baseline, i.e., prior to even starting any kind of cancer-directed therapy. This malnutrition at baseline persisted during the course of CRT and post-treatment. We identified groups of patients who more likely to be malnourished, including women, users of smokeless tobacco, and patients with buccal mucosa as the primary tumor site. Consuming smokeless tobacco has a detrimental effect on body weight, oral hygiene, and taste, leading to malnutrition [11]. National representative data from 2005 showed a gender bias where Indian women aged 15–49 years old systematically consumed less nutrient-rich foods [12]. A study from 2010 by Jager‐Wittenaar et al. assessed 29 patients undergoing chemotherapy or CRT 1 week before, 1 month after treatment, and 4 months after treatment; patients with adequate nutrient intake lost weight but to a lesser extent than patients with insufficient intake and had better recovery of lean body mass and weight post-treatment [13]. Intervention before the initiation of treatment to optimize nutritional status can improve outcomes during and post-treatment [14].

There was no statistically significant difference observed in 1-year survival rates based on the nutritional status. These findings suggest that nutritional status exerted no discernible impact on overall survival, progression-free survival, or locoregional relapse-free survival. Our findings were in contradiction to the existing data within this context. Several factors may have contributed to this discrepancy. Notably, all enrolled patients received regular dietary counseling and interventions from a dietitian, potentially ensuring adequate dietary support within our patient cohort. However, the lack of comprehensive documentation regarding dietary details in majority of patients introduced potential biases in the analysis.

Shivanna et al. have shown that nutritional indicators in patients with nasopharyngeal cancer were significantly lower after radiotherapy than the initial values before treatment [15]. The prognostic nutritional index (used as a biochemical index of nutritional status) revealed that the management of nutrition status did prolong survival [16]. Individualized dietary counseling on nutritional status and quality of life (QoL) resulted in better outcomes when compared to no standard nutritional advice in patients with head and neck squamous cell carcinoma [17]. Languis and colleagues conducted a systematic review of the effects of nutritional interventions on the nutrition, QoL and survival in patients with LAHNSCC receiving radiation or CRT. The interventions studied ranged from general dietary advice, individualized nutritional counselling, oral nutritional supplementation, nasogastric tube feeds, percutaneous endoscopic gastrostomy (PEG) feeding, and prophylactic PEG tube feeding. They found that individualized nutritional counselling positively impacted both the nutritional status and the patients’ QoL, as opposed to no counselling or general dietary advice. The data regarding oral dietary supplements, nasogastric tube and PEG tube feeding were inconsistent [18]. To the best of our knowledge, there have not been any studies on the nutritional status of Indian patients with LAHNSCC receiving CRT. Due to varying results and methodological limitations, a clear statement on the importance of nutritional counseling and intervention in patients with cancer is difficult currently [19].

This study encountered some limitations. Despite being a randomized prospective trial where nutritional status served as a secondary endpoint, our sample size was not powered to make any meaningful interpretation of the impact of nutritional status on oncologic outcomes. Moreover, a notable proportion of data was missing at each time point. Since the number of patients who filled out the nutrition forms was small, we were unable to delineate the impact of baseline nutritional status on outcomes based on the schedule of administration of cisplatin CRT. Additionally, the questionnaire was administered solely in one language, potentially introducing bias in patient comprehension and interpretation, despite a clinician’s assistance. Furthermore, being a single-center study with dietitian support, the findings may not be fully representative of the broader population within the country.

## Conclusion

Patients with good nutritional status before treatment initiation are more likely to maintain weight during curative CRT for head and neck cancer, underscoring the importance of pre-treatment implementation of medical nutrition therapy. Consequently, there is a compelling need for expanded research endeavors encompassing larger study populations to elucidate the multifaceted role of nutrition in patients with head-and-neck cancer and its impact on diverse treatment outcomes. Such investigations should incorporate regular dietary assessments and interventions aimed at enhancing patient compliance.

## Supplementary Information

Supplementary Material 1: Table S1. Distribution of various demographic and clinical factors across total nutrition scores between 3 weekly and weekly treatment groups compared using Chi-square test. Table S2. Comparison of total score of nutrition group (0-3 and >=4) at basline with the nutritional score at Day 21, Day 43 and FU 3. The P value is obtained from the McNemar test. Table S3. Lists the median followup using reverse Kaplan-Meier. The Kaplan meier method is used to obtain the Overall, progression free and loco-regional relapse free survival proportions estimates at 1 year with 95% CI. The log rank test is used to compare the survival between the patetinst with nutrition total scores 0-3 and >=4.

Supplementary Material 2.

## Data Availability

The authors confirm that the data supporting the findings of this study are available within the article.

## Abbreviations

LAHNSCC: Locally advanced head-and-neck squamous cell carcinoma
PG_SGA: Patient Generated Subjective Global Assessment
HNC: Head-and-neck cancer
NIS: Nutrition impact symptoms
CRT: Chemo-radiotherapy
SNTR: Scored nutritional triage recommendations
CI: Confidence interval
PFS: Progression-free survival
QoL: Quality of life
